# Impact of in-hospital SARS-CoV-2 infection on mortality and outcomes in patients admitted for heart failure: a nationwide analysis in Brazil

**DOI:** 10.3389/fcvm.2026.1723680

**Published:** 2026-03-24

**Authors:** Adriana Aparecida Bau, Camila Nicolela Geraldo Martins, Andréa Coy-Canguçu, Guilherme Cordeiro, Mauricio Longato, Otávio Rizzi Coelho, Thiago Quinaglia, Maria Luiza Moretti, Jose Roberto Mattos, Wilson Nadruz, Andrei Sposito, Múcio Tavares de Oliveira Junior, Michael Jerosch-Herold, Otávio Rizzi Coelho-Filho

**Affiliations:** 1 Department of Medicine, Discipline of Cardiology, University of Campinas School of Medical Sciences, Campinas, Brazil; 2 Catholic Pontifical University of Campinas Medical School, Campinas, Brazil; 3 Nodian, São Paulo, Brazil; 4 Heart Institute (InCor), University of São Paulo, Medical School, São Paulo, Brazil; 5 Non-Invasive Cardiovascular Imaging Program, Department of Radiology, Brigham and Women’s Hospital and Harvard Medical School, Boston, MA, United States

**Keywords:** COVID-19, heart failure, hospitalization, mortality, SARS-CoV-2

## Abstract

**Background:**

The effects of COVID-19 on patients hospitalized for heart failure (HF) remain underexplored, especially in middle-income countries. We investigated how in-hospital SARS-CoV-2 infection affected clinical outcomes and healthcare costs among heart failure (HF) patients in Brazil's public health system during the pandemic period, following its official global declaration in early 2020.

**Methods:**

We performed a retrospective study of 262,758 adults hospitalized for HF in public hospitals across Brazil from January-2020 to August-2021. Patients who acquired COVID-19 during hospitalization (*n* = 385) were compared to those who remained uninfected (*n* = 261,907). Demographics, comorbidities, clinical outcomes, and costs were analyzed. Cox regression models identified predictors of in-hospital mortality.

**Results:**

Patients with COVID-19 had longer hospital stays (median 7 vs. 5 days; *p* < 0.001), higher need for renal replacement therapy (6.5% vs. 2.6%; *p* < 0.001), and greater in-hospital mortality (26% vs. 13%; *p* < 0.001). Hospitalization costs were also higher in the COVID-19 group, both in total (median BRL 1,373 vs. BRL 836; *p* < 0.001) and per day (BRL 250 vs. BRL 226; *p* < 0.001). COVID-19 infection increased the risk of in-hospital death by 43% in univariate Cox regression (HR 1.43; 95% CI 1.14–1.68; *p* = 0.001), and remained independently associated with mortality in multivariable analysis (HR 1.38; 95% CI 1.14–1.68; *p* = 0.001). Kaplan–Meier analysis demonstrated significantly lower survival in the infected group (log-rank *p* < 0.001).

**Conclusion:**

In-hospital COVID-19 infection significantly worsens outcomes and increases healthcare costs among patients admitted for HF in Brazil. These findings underscore the need for preventive strategies, including vaccination and timely antiviral therapies, particularly within public health systems.

## Graphical Abstract

**Figure:**
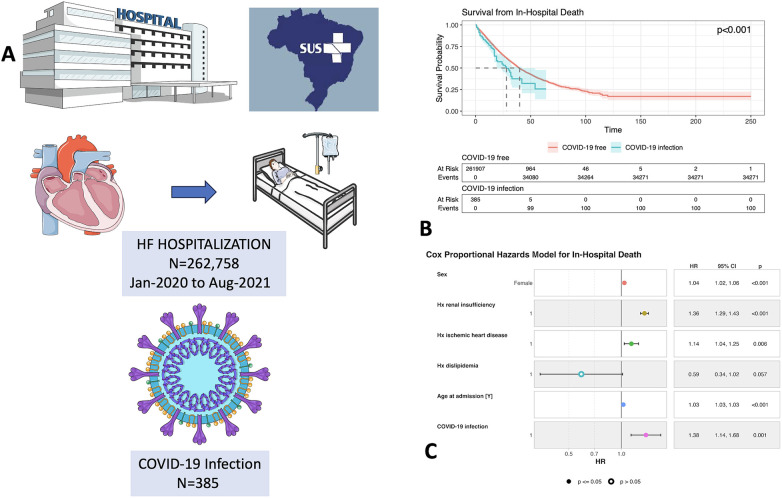
Impact of in-hospital COVID-19 infection on outcomes in patients hospitalized for heart failure. **(A)** Nationwide cohort from the Brazilian Unified Health System (SUS), including 262,758 heart failure (HF) hospitalizations between January 2020 and August 2021, of which 385 patients acquired COVID-19 during admission. **(B)** Kaplan–Meier survival analysis showing significantly lower in-hospital survival among patients who developed COVID-19 compared with those who remained COVID-19–free (log-rank *p* < 0.001). **(C)** Multivariable Cox proportional hazards model identifying independent predictors of in-hospital death, with COVID-19 infection associated with the highest risk after adjustment for clinical covariates. Image adapted from ***Servier Medical Art*** (https://smart.servier.com/), licensed under CC BY 4.0 (https://creativecommons.org/licenses/by/4.0/). HF, heart failure; SUS, Sistema Único de Saúde (Brazilian Unified Health System); COVID-19, coronavirus disease 2019; HR, hazard ratio; CI, confidence interval.

## Clinical perspective

In this nationwide study of over 260,000 hospitalizations for heart failure (HF) in Brazil's public health system, in-hospital acquisition of COVID-19 was independently associated with worse clinical outcomes and higher costs. Patients who developed SARS-CoV-2 infection had longer hospital stays, greater need for renal replacement therapy, and nearly double the risk of in-hospital death compared with non-infected patients. After multivariable adjustment, COVID-19 infection remained an independent predictor of mortality, with a 38% increased risk.

Vulnerable subgroups included patients with diabetes, hypertension, and those requiring dialysis, reflecting the greater susceptibility of individuals with multimorbidity and clinical complexity. These findings are consistent with international data, but particularly impactful in middle-income settings with constrained critical care resources.

Preventive strategies are urgently needed. Broader vaccine uptake, timely diagnosis, and improved access to antivirals could mitigate excess mortality and reduce the healthcare burden of COVID-19 in HF patients, who remain at disproportionate risk.

## Introduction

The COVID-19 pandemic profoundly disrupted healthcare delivery worldwide, particularly impacting patients with cardiovascular diseases (CVD). Brazil, a middle-income country with one of the largest public health systems globally, faced major challenges in managing the pandemic. The Human Development Index (HDI) varies substantially across regions, influencing access and outcomes. Previous studies documented a marked reduction in cardiovascular hospitalizations during the pandemic in Brazil, with a simultaneous increase in disease severity among hospitalized patients. Fagundes et al. (1) observed a marked decrease in CVD admissions, especially among younger individuals, women, and those from municipalities with lower HDI. However, hospitalized patients had a higher likelihood of requiring intensive care and had greater in-hospital mortality. SARS-CoV-2 infection is known to worsen outcomes among patients with cardiovascular conditions. Despite this, few studies evaluated nosocomial or in-hospital SARS-CoV-2 infection on HF patients, particularly in public health systems of low- and middle-income countries. Moreover, the real-world use of antiviral therapy has remained low, fewer than 35% of eligible high-risk patients in the U.S. have received antiviral treatment (2–4). Suboptimal COVID-19 vaccine uptake has also been reported (5).

In this study, we aimed to evaluate the impact of in-hospital SARS-CoV-2 infection on mortality, resource utilization, and healthcare costs in patients admitted for heart failure (HF) in Brazil's National Public Health System (SUS).

## Methods

This was a retrospective observational cohort, records-based study performed using data extracted from the SUS Hospital Information System (SIHSUS), a large database of de-identified data made publicly available by the Department of Informatics (DATASUS). DATASUS oversees the digital systems for the Brazilian Hospital Information System, which is responsible for delivering healthcare to approximately 78% of the Brazilian population who rely exclusively on public services. The study population consisted of adult patients (≥18 years) with a diagnosis of HF (ICD-10 code I50.x) hospitalized after the official declaration of the COVID-19 pandemic, between January 2020 and August 2021. COVID-19 diagnosis was based on the ICD-10 code assigned during hospitalization, as determined by the attending physician. This classification followed clinical guidelines in effect during the pandemic, which considered a combination of symptoms, physical findings, and PCR test results consistent with SARS-CoV-2 infection (6), and only patients with positive results during hospitalization were categorized in the COVID-19 infection group. Although COVID-19 diagnosis followed national guideline criteria, the structure and administrative nature of the database did not allow access to individual real-time PCR results or their exact timing. Nevertheless, all patients included in the analysis were admitted for decompensated heart failure, and none had clinical suspicion of COVID-19 or an ICD 10 diagnosis at the time of admission. The ICD-10 code for COVID-19 was assigned exclusively during hospitalization, indicating that SARS-CoV-2 infection was identified after admission and was not the reason for hospitalization. Follow-up duration was defined as the period between hospital admission and discharge or in-hospital death. Patients with missing data on admission date, discharge or death, age, sex, or hospital location were excluded.

This study adhered to the principles of the Declaration of Helsinki and was approved by the Institutional Review Board of the State University of Campinas (Of. CEP n° 117/2022), which waived the requirement for informed consent given the de-identified nature of publicly available data.

## Statistical analysis

Descriptive statistics were used to summarize the characteristics of the study population. Continuous variables were expressed as medians and interquartile ranges (IQR), and categorical variables as absolute values and percentages. Comparisons between COVID-19-infected and non-infected groups were made using the Mann–Whitney *U*-test for continuous variables and the Chi-square or Fisher's exact test for categorical variables. To explore factors associated with SARS-CoV-2 infection during hospitalization, we performed logistic regression analyses. COVID-19 infection was treated as a binary outcome variable. Univariate models were initially constructed to screen for potential predictors, including demographic variables (age, sex), comorbid conditions (e.g., hypertension, diabetes, ischemic heart disease), and the need for dialysis during admission. Variables with a *p*-value <0.10 in univariate analysis were subsequently entered into a multivariable logistic regression model. The effects of predictors on COVID-19 infection are reported as odds ratios (OR) with corresponding 95% confidence intervals (CI). Survival curves for in-hospital death were generated using the Kaplan–Meier method, and differences between groups were assessed using the log-rank test. To evaluate factors associated with in-hospital mortality, Cox proportional hazards (PH) models were used. Predictors in the Cox PH model were chosen by stepwise model selection based on the AIC (Akaike Information Criterion). Assumptions of proportionality were tested using Schoenfeld residuals and visually inspected with log-minus-log plots. Given the limitations of administrative data in defining the precise timing of in-hospital events, the multivariable models were intentionally restricted to baseline and pre-existing characteristics present at admission. Variables that could occur during hospitalization were therefore excluded and replaced by clinically relevant historical conditions not subject to temporal ambiguity. All Cox proportional hazards models were re-estimated using this refined approach. Statistical significance was defined as *p* < 0.05. The probability of in-hospital death was additionally analyzed using logistic regression, meaning without consideration of the follow-up time, or reliance on the time of COVID-19 infection. This model included the same predictors as the Cox PH model. All analyses were performed using R software (version 4.3.1).

## Results

### Clinical and demographic characteristics

Out of 262,758 patients hospitalized for HF, 385 (0.15%) were diagnosed with SARS-CoV-2 infection during hospitalization. Table 1 summarizes the clinical and demographic characteristics of the COVID-19 infected and non-infected groups. The median age was similar across groups (69 years), though a higher proportion of COVID-19 patients were female (53% vs. 48%, *p* = 0.038). Comorbidities were more frequent among COVID-19 patients, including diabetes (8.6% vs. 2.1%, *p* < 0.001), hypertension (18% vs. 4.9%, *p* < 0.001), atrial fibrillation (2.1% vs. 0.7%, *p* = 0.008), and chronic kidney disease (4.4% vs. 1.9%, *p* < 0.001). During the duration of the Covid-19 pandemic, the percentage of HF patients with Covid-19 infection varied significantly between the federal states of Brazil (chi-square *p*-value: <0.001) and was highest in Amapá in the Amazon basin (0.84%), followed by Tocantins (0.53%) and São Paulo (0.42%), as illustrated in Supplementary Figure S1.

**Table 1:** Baseline characteristics of HF patients with and without COVID-19 infection.

Variable	COVID-19 free (*N* = 261,907)^a^	COVID-19 infection (*N* = 385)^a^	*p*-value^b^
Age at admission	69 (58, 78)	69 (59, 77)	0.744
Female sex	124,964 (48%)	204 (53%)	0.038
Race			
White	99,869 (48%)	169 (55%)	
“Parda”	89,052 (43%)	111 (36%)	
Black	13,819 (6.6%)	21 (6.8%)	
Asian	5,573 (2.7%)	6 (2.0%)	
Indigenous	278 (0.1%)	0 (0%)	
Days of hospitalization	5.0 (3.0, 9.0)	7.0 (4.0, 14)	<0.001
In-hospital death	34,271 (13%)	100 (26%)	<0.001
Hx diabetes	5,564 (2.1%)	33 (8.6%)	<0.001
Hx hypertension	12,851 (4.9%)	69 (18%)	<0.001
Hx dyslipidemia	133 (<0.1%)	1 (0.3%)	0.179
Hx atrial fibrillation/flutter	1,922 (0.7%)	8 (2.1%)	0.008
Hx stroke	789 (0.3%)	3 (0.8%)	0.112
Hx renal insufficiency	4,889 (1.9%)	17 (4.4%)	<0.001
RRT	6,858 (2.6%)	25 (6.5%)	<0.001
Dialysis type			
None	255,068 (97%)	360 (94%)	
Continuous dialysis	949 (0.4%)	1 (0.3%)	
Peritoneal dialysis	103 (<0.1%)	1 (0.3%)	
Intermittent dialysis	5,787 (2.2%)	23 (6.0%)	
Renal Replacement Therapy	26 (<0.1%)	1 (0.3%)	0.039
Obstructive pulmonary disease	2,288 (0.9%)	3 (0.8%)	>0.999
Chagas’ disease	327 (0.1%)	1 (0.3%)	0.383
Hx coronary bypass	3 (<0.1%)	0 (0%)	>0.999
In-hospital Heart Tx	251 (<0.1%)	0 (0%)	>0.999
Total hospitalization cost (BRL)	836 (715, 1,377)	1,373 (861, 3,884)	<0.001
Cost per day (BRL)	226 (122, 358)	250 (129, 498)	<0.001
Year of admission			
2020	128,322 (49%)	215 (56%)	0.007
2021	133,585 (51%)	170 (44%)	
Geographic location			
Central-West	17,567 (6.7%)	20 (5.2%)	<0.001
Northeast	56,668 (22%)	27 (7.0%)	
North	13,778 (5.3%)	14 (3.6%)	
Southeast	112,094 (43%)	298 (77%)	
South	61,800 (24%)	26 (6.8%)	

^a^ Median (IQR); *n* (%).

^b^ Kruskal–Wallis rank sum test; Pearson's Chi-squared test; Fisher's exact test.

### Clinical predictors of in-hospital SARS-CoV-2 infection

**Figure 1:**
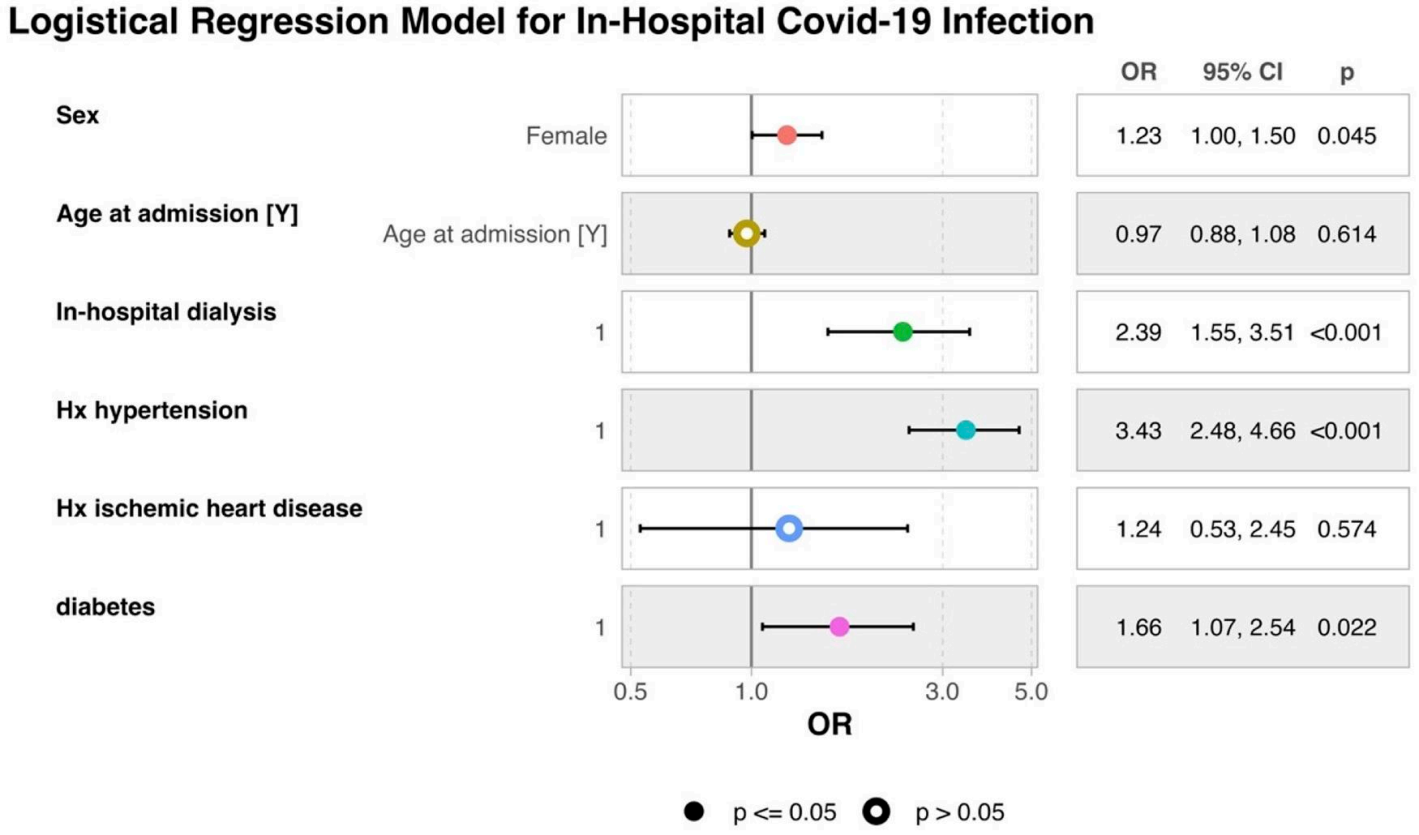
Multivariable logistic regression model of risk factors for SARS-CoV-2 infection acquired during heart failure hospitalization. OR, odds ratio; CI, confidence interval. Forest plot of multivariable logistic regression showing adjusted odds ratios (ORs) with 95% confidence intervals for factors associated with acquiring COVID-19 during HF hospitalization in Brazil's SUS (*N* = 262,758; COVID-19 infection *n* = 385; no infection *n* = 261,907). Covariates were selected from univariate screening (*p* < 0.10) and included age, sex, hypertension, diabetes, atrial fibrillation/flutter, chronic kidney disease, ischemic heart disease, and the need for renal replacement therapy during the index admission. OR > 1 indicates higher odds of nosocomial infection. COVID-19 was defined by ICD-10 coding with PCR confirmation during hospitalization per contemporaneous clinical guidelines. CI, confidence interval; ICD-10, international classification of diseases, 10th revision.

The association between baseline clinical features and the risk of COVID-19 infection during hospitalization for HF was assessed. Figure 1 presents the multivariable logistic regression model, including selected clinical and demographic variables of interest. Female sex was associated with a modest but statistically significant increase in the odds of in-hospital SARS-CoV-2 infection (OR 1.23; 95% CI, 1.00–1.50; *p* = 0.045). In contrast, age at admission was not independently associated with risk of infection (OR 0.97; 95% CI, 0.88–1.08; *p* = 0.614). Among comorbidities, a prior diagnosis of hypertension was strongly associated with COVID-19 acquisition (OR 3.43; 95% CI, 2.48–4.66; *p* < 0.001), as was diabetes (OR 1.66; 95% CI, 1.07–2.54; *p* = 0.022). Patients who required dialysis during their hospitalization were also at higher risk (OR 2.39; 95% CI, 1.55–3.51; *p* < 0.001). By contrast, a history of ischemic heart disease did not remain significant after adjustment (OR 1.24; 95% CI, 0.53–2.45; *p* = 0.574). These findings suggest that individuals with greater comorbidity burden and clinical complexity may be more vulnerable to acquiring SARS-CoV-2 infection while hospitalized. The association with dialysis, in particular, may reflect increased exposure due to procedural needs and prolonged hospitalization, rather than underlying renal dysfunction alone.

### Clinical outcomes of hospitalized HF patients with concomitant COVID-19 infection

Among patients hospitalized with HF, those who acquired a confirmed SARS-CoV-2 infection during their stay had significantly worse clinical outcomes. These individuals experienced longer hospitalizations (median 7 vs. 5 days; *p* < 0.001), a higher need for renal replacement therapy (6.5% vs. 2.6%; *p* < 0.001), and markedly higher in-hospital mortality rates (26% vs. 13%; *p* < 0.001). Additionally, the financial burden was substantially greater in this group, with higher median total hospitalization costs (BRL 1,373 vs. BRL 836; *p* < 0.001) and higher median daily costs (BRL 250 vs. BRL 226; *p* < 0.001).

**Figure 2:**
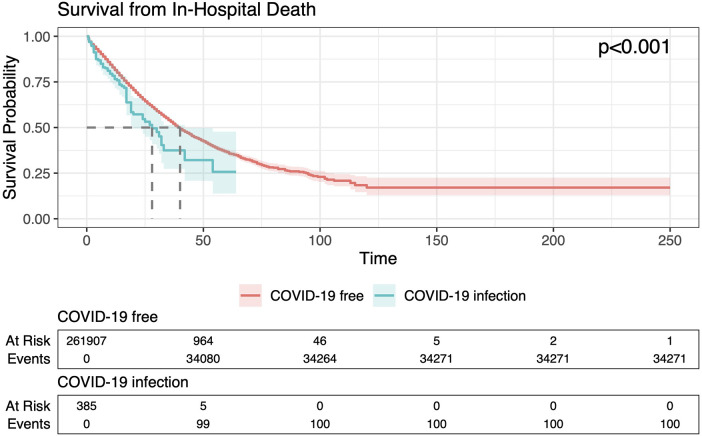
Kaplan–Meier survival curve comparing in-hospital mortality among HF patients with and without COVID-19 infection. Kaplan–Meier curves presenting probability of survival from admission through discharge or in-hospital death, comparing patients who acquired COVID-19 during the hospitalization vs. those who did not. Time scale is days since hospital admission. Shaded bands denote 95% CIs. Survival curves diverge early with significantly lower survival in infected patients (log-rank *p* < 0.001). Number-at-risk tables refer to patients remaining under observation at each time point. CI, confidence interval.

**Figure 3:**
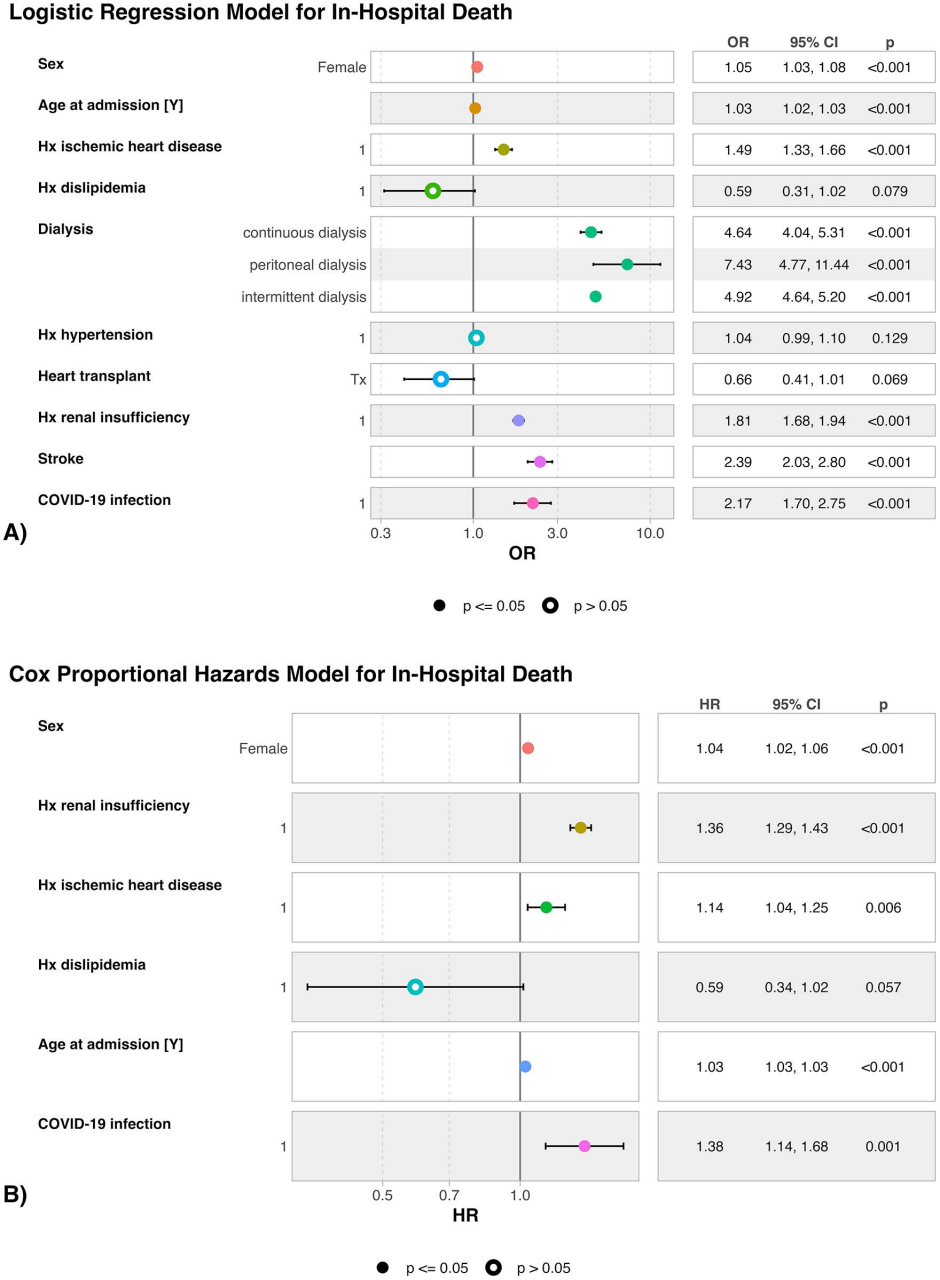
Logistic regression and Cox proportional hazards models for in-hospital death. (A) Multivariable logistic regression model displaying adjusted ORs (95% CIs) for in-hospital death; (B) Cox proportional hazards model displaying adjusted hazard ratios (HRs) (95% CIs). Covariates in the logistic regression model include age, sex, ischemic heart disease, dyslipidemia, renal replacement therapy, hypertension, previous heart transplant, chronic kidney disease, stroke, and in-hospital COVID-19 infection. Model selection for the Cox analysis used stepwise AIC; proportional hazards assumptions were assessed with Schoenfeld residuals. OR or HR >1 indicates higher odds/hazard of death. AIC, akaike information criterion; CI, confidence interval; HR, hazard ratio; OR, odds ratio.

Univariate Cox regression analysis for in-hospital death (Supplementary Table S1) shows that COVID-19 infection was associated with a 43% increase in the hazard of in-hospital mortality (HR 1.43; 95% CI: 1.17–1.74; *p* < 0.001). Other univariate associations with death included older age at admission (HR per year 1.03; 95% CI: 1.03–1.03; *p* < 0.001), female sex (HR 1.13; 95% CI: 1.11–1.16; *p* < 0.001), history of ischemic heart disease (HR = 1.19; 95% CI: 1.08–1.31; *p* < 0.001), and history of renal insufficiency (HR 1.40; 95% CI: 1.33–1.47; *p* < 0.001). Kaplan–Meier survival curves (Figure 2, graphical abstract) illustrate a clear early divergence in survival probability between infected and non-infected patients, with those affected by COVID-19 exhibiting significantly lower survival throughout the hospitalization period. By day 10, survival probability was already substantially reduced among infected patients. The log-rank test confirmed the statistical significance of these differences (*p* < 0.001). In the multivariable Cox model (Figure 3B, graphical abstract and Supplementary Table S1), adjusted for age, sex, comorbidities, and history of renal insufficiency, in-hospital COVID-19 infection remained independently associated with higher mortality (HR 1.38; 95% CI 1.14–1.68; *p* = 0.001), along with older age at admission (HR per year 1.03; 95% CI: 1.03–1.03; *p* < 0.001), female sex (HR 1.04; 95% CI, 1.02–1.06; *p* < 0.001), history of ischemic heart disease (HR 1.14; 95% CI: 1.04, 1.25; *p* < 0.01), and history of renal insufficiency (HR 1.36; CI: 1.29–1.43; *p* < 0.001). The odds of in-hospital death were associated in a multi-variate logistic regression model (Figure 3A) with female sex (OR 1.05; 95% CI: 1.03, 1.08; *p* < 0.001), history of ischemic heart disease (OR 1.49; 95% CI: 1.33–1.66; *p* < 0.001), renal replacement therapy (*p* < 0.001 for all types of dialysis), and Covid-19 infection (OR 2.17; 95% CI: 1.70–2.75; *p* < 0.001). It trended lower with previous heart transplantation (OR 0.66; 95% CI: 0.41–1.10, *p* = 0.068).

## Discussion

This large-scale nationwide analysis represents the first dedicated investigation into the clinical and economic burden of in-hospital SARS-CoV-2 infection among patients admitted for HF within Brazil's public healthcare system. Our findings clearly demonstrate that a confirmed COVID-19 infection acquired during hospitalization is independently associated with markedly worse clinical outcomes, including a more than 40% higher risk of in-hospital mortality (univariate HR: 1.43, CI: 1.17, 1.74, *p* < 0.001, even after adjustment for relevant comorbidities and demographic variables (Adjusted OR: 2.17; 95% CI: 1.70, 2.75, *p* < 0.001; Adjusted HR: 1.38, CI: 1.14–168, *p* = 0.001, Figures 2, 3). These patients also experienced longer hospital stays, increased need for renal replacement therapy, and incurred substantially higher healthcare costs. Notably, the risk of acquiring SARS-CoV-2 during hospitalization was significantly higher among patients with greater clinical complexity—particularly those with diabetes, hypertension, and those requiring dialysis (Figure 1)—who also experienced the poorest outcomes once infected. Prior studies identified diabetes (7) and hypertension (8) as risk factors that increase the susceptibility to Covid-19 infection, due to compromised immune function. Together, these findings underscore the disproportionate impact of COVID-19 on an already vulnerable population and reveal a considerable strain on public healthcare resources.

Our results align with prior evidence from large-scale analyses in other countries. For instance, in a U.S. study of patients hospitalized with HF, Isath et al. (9) reported significantly higher mortality, longer length of stay, and greater use of intensive care among those co-infected with COVID-19. This cross-national consistency reinforces the notion that HF patients represent a high-risk group when exposed to SARS-CoV-2 infection diagnosed during hospitalization, irrespective of healthcare system context. These findings highlight the urgent need for preventive strategies, particularly in low- and middle-income settings with limited critical care capacity. Accumulating evidence from meta-analyses, adjudicated outcome registries, and large population-based studies has demonstrated a consistent association between SARS-CoV-2 infection and cardiovascular events, including myocardial infarction, heart failure, stroke, thromboembolic complications, and cardiovascular mortality. A meta-analysis by Shoar et al. demonstrated significantly higher rates of cardiovascular events and adverse cardiovascular biomarkers among non-survivors compared with survivors of COVID-19 (10). In addition, a large study with physician-adjudicated outcomes by Bikdeli et al. showed a substantial burden of cardiovascular events following COVID-19 infection and a modifying effect of vaccination status (11). Furthermore, a nationwide population-based longitudinal study by Spetz et al. demonstrated increased short- and long-term cardiovascular risk after COVID-19 infection, even among individuals with mild disease, with particularly elevated risks for thromboembolic events (12). Together, these data place our findings within the broader and well-established cardiovascular impact of COVID-19 and support the biological plausibility of the adverse outcomes observed among patients hospitalized for heart failure who acquire SARS-CoV-2 infection during hospitalization.

Nosocomial SARS-CoV-2 infection has been reported across different hospitalized populations and, although its incidence varies according to diagnostic criteria and local epidemiological context, it has consistently been associated with worse clinical outcomes (13, 14). In a large nationwide Spanish study, nosocomial COVID-19 accounted for 4.8% of hospitalized cases and was associated with a markedly higher in-hospital mortality compared with community-acquired infection, 39.1% vs. 19.2%, remaining an independent predictor of death after multivariable adjustment (13). Notably, acute heart failure was substantially more frequent among patients with nosocomial infection, occurring in 20.0% of cases compared with 8.4% in those with community-acquired COVID-19, underscoring the vulnerability of patients with cardiovascular disease who acquire SARS-CoV-2 during hospitalization (13). Observations from other hospital-based cohorts indicate that nosocomial acquisition occurred even in institutions with structured infection-control measures, highlighting that hospital-acquired COVID-19 remained a relevant clinical problem during the pandemic (15). Brazilian data provide additional context. National epidemiological analyses identified nosocomial transmission in approximately 2.95% of hospitalized COVID-19 cases during early pandemic waves (16), while the Brazilian COVID-19 Registry reported nosocomial infections as in-hospital complications in 13.1% of admissions, within a cohort characterized by high mortality rates (17). Despite this growing body of evidence, to the best of our knowledge, no studies have been specifically designed to evaluate nosocomial SARS-CoV-2 infection among patients admitted primarily for acute decompensated heart failure. Prior investigations have either assessed heterogeneous hospitalized populations with nosocomial COVID-19 or examined outcomes of COVID-19 in patients with heart failure, without isolating hospital-acquired infection during HF hospitalization as the exposure of interest (13, 14, 18). Within this context, the present study adds new information by specifically addressing the incidence and prognostic impact of in-hospital SARS-CoV-2 infection in patients hospitalized for heart failure in a large public healthcare system.

Although the incidence of SARS-CoV-2 infection diagnosed during hospitalization for heart failure was low in our cohort, its clinical consequences were substantial, highlighting the importance of preventive strategies within hospital settings. Early experience during the pandemic showed that prompt case identification, isolation, and structured management of exposed patients were central to limiting in-hospital transmission (19). Several pharmacologic and non-pharmacologic approaches have since been explored, including pre-exposure prophylaxis with long-acting monoclonal antibodies (20), post-exposure antiviral therapy (21), early antiviral treatment (22), and topical prophylaxis aimed at reducing viral acquisition (23). However, none of these strategies has been specifically evaluated in patients admitted for acute heart failure, and their effectiveness in this population remains uncertain. In this setting, consistent application of standard infection-control measures after exposure remains the most reliable approach, while other preventive strategies should be viewed as potentially relevant but unproven adjuncts. Importantly, vaccination has been shown to mitigate these adverse effects. Johnson et al. (24) demonstrated that fully vaccinated HF patients, particularly those who received booster doses, had significantly lower risks of hospitalization, ICU admission, and death compared to their unvaccinated counterparts. Despite this, vaccine uptake remains below optimal levels in many countries. Recent CDC reports indicate subpar adherence to vaccination guidelines among adults in the U.S. (5), and similar patterns likely apply to Brazil. In addition, uptake of antiviral therapy among high-risk outpatients has remained below 35%, despite guideline-based recommendations (2–4). The underutilization of these proven interventions may have contributed to the unfavorable outcomes observed in our cohort.

Our findings thus reinforce the urgency of improving vaccine coverage and antiviral access for HF patients, particularly those in public health systems such as Brazil's SUS, which provides care to the majority of the population. Pharmacologic treatments, including oral antivirals, have been shown to reduce the risk of complications when initiated early (21, 25), especially in clinically vulnerable groups such as those with diabetes or kidney disease. These measures are vital not only to reduce mortality but also to alleviate the broader healthcare burden exacerbated by COVID-19 in patients with chronic cardiovascular conditions. Unfortunately, vaccine uptake remains suboptimal in Brazil (26), and access to evidence-based treatments for high-risk inpatients continues to be limited, despite their proven benefits.

Moreover, vaccination has come to represent far more than a tool to prevent infection. The 2025 statement from the European Society of Cardiology on “Vaccination as a new form of cardiovascular prevention” highlights that immunization against respiratory pathogens such as influenza, pneumococcus, and SARS-CoV-2 should be viewed as an essential part of cardiovascular care (27). This perspective stems from a growing body of evidence showing that infections can trigger inflammatory and thrombotic cascades, leading to plaque destabilization, myocardial injury, and decompensation of chronic heart failure. For patients with heart failure or coronary artery disease, protection against SARS-CoV-2 has been shown to reduce the risk of hospitalization, cardiovascular complications, and death. Broader vaccination coverage helps reduce hospital admissions, preserve intensive care capacity, and ease the financial pressure on public health systems. In countries such as Brazil, where the public sector carries most of the burden of cardiovascular care, incorporating structured vaccination strategies into heart failure management could bring meaningful clinical and economic benefits. As underscored by the European Society of Cardiology (27), vaccination should now be regarded as a natural extension of established cardiovascular prevention. Alongside lipid control, blood pressure management, and smoking cessation, immunization offers an opportunity to prevent avoidable complications and to protect patients whose vulnerability extends beyond the traditional boundaries of cardiovascular disease.

This nationwide analysis provides real-world evidence that SARS-CoV-2 infection identified during hospitalization for HF is independently associated with higher in-hospital mortality, longer length of stay, and increased healthcare costs. By focusing on patients admitted for HF within a large public health system, this study addresses a relevant gap in the literature, which has predominantly examined heterogeneous hospitalized populations or community-acquired infection. Although information on vaccination status and antiviral therapy was not available in the present dataset, evidence from other settings indicates that preventive strategies, including vaccination, early diagnosis, and access to effective antiviral treatment, reduce COVID-19 severity and mortality. In this broader public health context, our findings support the hypothesis that strengthening such measures may help mitigate the excess risk observed among hospitalized patients with HF. These statements are intended as contextual public health considerations rather than direct inferences from the current data.

## Limitations

Despite the strengths of the present study, including its national scope and the use of real-world data from Brazil's public health system, certain limitations should be acknowledged. First, as the analysis was based on administrative data from the SIHSUS registry, it lacks granular clinical and laboratory information, such as left ventricular ejection fraction, biomarkers, or details on pharmacologic therapy, which could provide a deeper understanding of disease severity and treatment response. Second, comorbidities were identified using ICD-10 codes from an administrative database and therefore represent coded comorbidities, rather than their true clinical prevalence. Validation studies in cardiovascular populations have demonstrated systematic under-ascertainment of chronic conditions in administrative data, particularly for comorbidities not central to the index hospitalization, despite low false-positive rates (28). As a result, the prevalence of some comorbidities in our cohort may be underestimated, which may attenuate the estimated associations between individual comorbidities and outcomes in multivariable models. However, prior methodological work has shown that administrative data–based comorbidity measures remain valid for risk adjustment and mortality prediction in large population-based studies when applied consistently across groups (28). Because any undercoding is expected to be largely non-differential between patients who did and did not acquire SARS-CoV-2 during hospitalization, this limitation does not undermine the main conclusions of the study. Third, although only patients with SARS-CoV-2 infection, as reflected by ICD coding that aligns with clinical diagnosis consistent with guidelines, were included in the COVID-19 group, undiagnosed cases, especially during the early phase of the pandemic when testing was less widely available, cannot be entirely excluded. Fourth, the nature of the dataset does not allow for precise determination of the timing of infection during hospitalization; although the exact date of infection is unavailable, the infection is known to have occurred during the hospital stay for survival analysis, nor does it capture the evolving nature of viral variants or changes in treatment protocols over time. Therefore, an analysis of the probability of in-hospital death with a logistic regression model was added. Hazard ratios from a Cox PH model and odds ratios from a logistic regression model capture different aspects of the relationship between predictors and outcome. The comparison of the results from these two types of models therefore focused direction of the effects (e.g., whether a predictor increases or decreases the hazard/odds). We found consistency of the effect directions, including from Covid-19 infection, a higher hazard of in-hospital death in the survival analysis, and a larger odds of in-hospital death from the logistic regression analysis. Moreover, the administrative database lacks information on vaccination status, disease severity, COVID-19 therapies, and viral variants, limiting causal inference and assessment of effect modification. Accordingly, statements regarding prevention or treatment should be interpreted as hypotheses rather than direct findings. Fifth, as with all retrospective observational studies, the possibility of residual confounding remains, despite statistical adjustment for measured covariates. Finally, the administrative nature of the database does not allow precise determination of the timing of SARS-CoV-2 acquisition relative to hospital admission, as testing dates and present on admission indicators are unavailable. Therefore, some infections identified during hospitalization may reflect pre-admission exposure with asymptomatic or presymptomatic incubation, and the findings should be interpreted as the prognostic impact of SARS-CoV-2 infection identified during heart failure hospitalization rather than confirmed nosocomial transmission.

## Conclusion

In patients hospitalized for HF, acquiring COVID-19 during the hospital stay was associated with a substantially higher risk of complications, including death, longer hospitalizations, greater use of life-support therapies, and higher medical costs. Patients with diabetes, hypertension, or requiring dialysis appeared more susceptible to nosocomial infection, highlighting the importance of targeted preventive measures. These findings highlight how severely a COVID-19 infection can affect individuals already facing the challenges of heart failure, especially in public health systems with limited resources. This study reinforces the urgent need for protective strategies in this vulnerable population. Improving vaccination coverage, timely diagnosis, and access to antiviral therapies may reduce preventable harm.

## Data Availability

The raw data analyzed in this study are publicly available, de-identified administrative data from the DATASUS/SIHSUS database of the Brazilian Ministry of Health and can be freely accessed at https://datasus.saude.gov.br/informacoes-de-saude-tabnet/. For the present study, the authors performed data extraction, cleaning, and restructuring of the publicly available DATASUS data to generate curated analytical datasets required for the statistical analyses. These processed datasets do not contain information beyond the original public data and can be made available upon reasonable request to the corresponding author.

## Supplementary material

Supplementary Figure S1 Percentage of patients admitted for heart failure with Covid-19 infection by Federal State. Map of Brazilian states showing the proportion of patients admitted for heart failure who developed COVID-19 during hospitalization (January 2020–August 2021). The highest frequencies were observed in Amapá, Tocantins, and São Paulo. Differences between states were statistically significant (chi-square test, *p* < 0.001).

Supplementary Table S1 Hazard Ratios for In-Hospital Mortality from Univariate and Stepwise-Selected Multivariable Cox Models.
