# Supplementary material for: Impact of in-hospital SARS-CoV-2 infection on mortality and outcomes in patients admitted for heart failure: a nationwide analysis in Brazil
Source: Front Cardiovasc Med. 2026 Mar 24;13:1723680. doi: 10.3389/fcvm.2026.1723680 (PMC13079563; doi:10.3389/fcvm.2026.1723680)
Supplement: Supplementary Figure S1 — Percentage of patients admitted for heart failure with Covid-19 infection by Federal State. Map of Brazilian states showing the proportion of patients admitted for heart failure who developed COVID-19 during hospitalization (January 2020–August 2021). The highest frequencies were observed in Amapá, Tocantins, and São Paulo. Differences between states were statistically significant (chi-square test, p < 0.001). [file Image1.pdf]

Supplemental Figure 1: Percentage of Patients Admitted for Heart Failure with Covid-19 Infection by Federal State

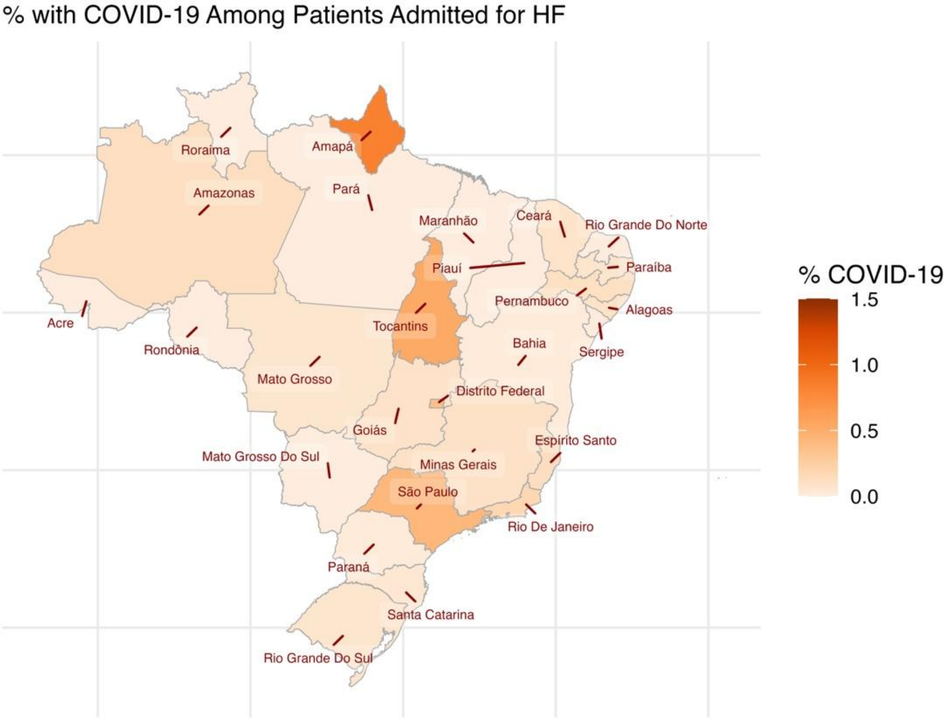

<u>Suppl Figure 1 (legend):</u> Map of Brazilian states showing the proportion of patients admitted for heart failure who developed COVID-19 during hospitalization (January 2020–August 2021). The highest frequencies were observed in Amapá, Tocantins, and São Paulo. Differences between states were statistically significant (chi-square test, $p<0.001$).
