# Supplementary material for: Impact of in-hospital SARS-CoV-2 infection on mortality and outcomes in patients admitted for heart failure: a nationwide analysis in Brazil
Source: Front Cardiovasc Med. 2026 Mar 24;13:1723680. doi: 10.3389/fcvm.2026.1723680 (PMC13079563; doi:10.3389/fcvm.2026.1723680)
Supplement: Supplementary Table S1 — Hazard Ratios for In-Hospital Mortality from Univariate and Stepwise-Selected Multivariable Cox Models. [file Table1.docx]

**Supplemental Table 1**: Hazard Ratios for In-Hospital Mortality from Univariate and Stepwise-Selected Multivariable Cox Models.

| **Cox PH Models** | Univariate | | | Multivariate | | |
| --- | --- | --- | --- | --- | --- | --- |
| **Characteristic** | **HR** | **95% CI** | **p-value** | **HR** | **95% CI** | **p-value** |
| Female sex | 1.13 | 1.11, 1.16 | **<0.001** | 1.04 | 1.02, 1.06 | **<0.001** |
| Hx renal insufficiency | 1.40 | 1.33, 1.47 | **<0.001** | 1.36 | 1.29, 1.43 | **<0.001** |
| Hx ischemic heart disease | 1.19 | 1.08, 1.31 | **<0.001** | 1.14 | 1.04, 1.25 | **0.006** |
| Hx dislipidemia | 0.61 | 0.36, 1.06 | 0.079 | 0.59 | 0.34, 1.02 | 0.057 |
| Age at admission [Y] | 1.03 | 1.03, 1.03 | **<0.001** | 1.03 | 1.03, 1.03 | **<0.001** |
| Covid-19 infection | 1.43 | 1.17, 1.74 | **<0.001** | 1.38 | 1.14, 1.68 | **0.001** |
| Abbreviations: CI = Confidence Interval, HR = Hazard Ratio | | | | | | |
| No. Obs. = 34,371 | | | | | | |
